# Strain Rate and Stress Amplitude Effects on the Mechanical Behavior of Carbon Paste Used in the Hall–Héroult Process and Subjected to Cyclic Loadings

**DOI:** 10.3390/ma15031263

**Published:** 2022-02-08

**Authors:** Zahraa Kansoun, Hicham Chaouki, Donald Picard, Julien Lauzon-Gauthier, Houshang Alamdari, Mario Fafard

**Affiliations:** 1 Department of Civil and Water Engineering, NSERC/Alcoa Industrial Research Chair MACE3, Aluminium Research Centre—REGAL, Université Laval, Quebec, QC G1V 0A6, Canada; mario.fafard.2@ulaval.ca; 2 SAFI Quality Software Inc., Quebec, QC G1X 1S7, Canada; hicham.chaouki.1@ulaval.ca; 3 Eddify Technologies Company, Quebec, QC G1P 0B3, Canada; dpicard@eddyfi.com; 4 Continuous Improvement Smelting Technology, Alcoa, Deschambault-Grondines, QC G0A 1S0, Canada; julien.lauzon-gauthier@alcoa.com; 5 Department of Mining, Metallurgical and Materials Engineering, NSERC/Alcoa Industrial Research Chair MACE3, Aluminium Research Centre—REGAL, Université Laval, Quebec, QC G1V 0A6, Canada; houshang.Alamdari@gmn.ulaval.ca

**Keywords:** carbon paste, cyclic compaction, strain rate, stress amplitude, unloading level

## Abstract

Carbon products such as anodes and ramming paste must have well-defined physical, mechanical, chemical, and electrical properties to perform their functions effectively in the aluminum electrolysis cell. The physical and mechanical properties of these products are assigned during the shaping procedure in which compaction stresses are applied to the green carbon paste. The optimization of the shaping process is crucial to improving the properties of the carbon products and consequently to increasing the energy efficiency and decreasing the greenhouse gas emissions of the Hall–Héroult process. The objective of this study is to experimentally investigate the effect(s) of the strain rate, of the stress maximum amplitude, and of the unloading level on the behavior of a green carbon paste subjected to cyclic loading. To this end, experiments consisting of (1) cyclic compaction tests at different maximum stress amplitudes and strain rates, and (2) cyclic compaction tests with different unloading levels were carried out. The study obtained the following findings about the behavior of carbon paste subjected to cyclic loads. The strain rate in the studied range had no effect either on the evolution of the permanent strain as a function of the cycle number, nor on the shape of the stress–strain hysteresis during the cyclic loading. Moreover, samples of the same density that had been subjected to different maximum stress amplitudes in their loading history did not have the same shape of the stress–strain curve. On the other hand, despite having different densities, samples subjected to the same number of cycles produce the same stress–strain curve during loading even though they were subjected to different maximum stress amplitudes in their loading histories. Finally, the level of unloading during each cycle of a cyclic test proved significant; when the sample was unloaded to a lower level of stress during each cycle, the permanent strain as a function of the cycle number was higher.

## 1. Introduction

The Hall–Héroult process is used worldwide to produce primary aluminum. This process aims to transform the raw material alumina (Al_3_O_3_) into liquid aluminum (Al) in an electrolysis cell operating at high temperatures (920–980 °C) [1]. A direct current travels the electrolysis cell downwards; it passes through the carbon anode and the cryolite bath where the alumina is dissolved, and finally through the cathode blocks. A voltage drop occurs when the electrical current travels the cell, partially due to Joule’s effect related to the resistance of the cell’s components. For a typical electrolysis cell, the voltage drops at the anode and the cathode levels account for 8.05% and 6.90%, respectively, of the total cell voltage [1]. To decrease the overall cost of aluminum production, reducing energy consumption has become critical [1]. 

The carbon anode is consumed gradually during the electrolysis and is replaced every 25–35 days. The anode quality directly affects the electrolysis cost [2]. A poorly compacted anode will have a high electrical resistivity, a high chemical reactivity, and low mechanical properties [3]. This leads to a higher voltage drop at the anode level, and an increase in the consumption rate of the carbon and the GHG emissions. Under such conditions, the anode may suffer from critical ruptures during the manipulation or the electrolysis process, making it unusable [4]. 

At the base of the electrolysis cell, the ramming paste acts as a joint between the cathode blocks and as a peripheral seam of the cell. The role of the ramming paste is to absorb the thermal expansion of the cathodic blocks during the cell operation and to prevent the infiltration of any liquids into the refractory lining of the electrolysis cell. A poorly compacted ramming paste will suffer from shrinkage and cracking during the cell preheating and operation [5]. Consequently, the risk of liquid infiltration will increase, which can shorten the cell life span [5]. 

Both the anode block and the ramming paste are made of carbon aggregates (e.g., calcined coke, calcined anthracite) and a binder. Most often, the binder used in the electrolysis cell is the coal tar pitch, even though there is a general trend to replace it in the ramming paste with green pitches that do not pose a health risk for the employees that handle the carbon paste. In addition to these raw materials, the ramming paste contains softeners which decrease the viscosity of the pitch and make the paste malleable at room temperature. While the ramming paste is being rammed in-situ between and around the cathode blocks at room temperature, the anode block is compacted and prebaked prior to its insertion in the cell. The anode raw materials are mixed and then compacted at high temperatures that depend on their properties and the anode paste recipe and which are around 178 °C and 150 °C, respectively [6]. Nowadays, most of the prebaked anode suppliers and aluminum smelters are shaping the anode blocks by vibro-compaction. 

An optimization of the green anode and the ramming pastes compaction processes is of critical importance as a remedy for the problems related to poor compaction. Such optimization requires a deep understanding of the carbon paste’s behavior under cyclic compaction forces. Unfortunately, this behavior is still ill-understood and under-studied. 

Several works have studied the effects of the raw material quality and proportions [7,8,9,10,11,12,13,14,15,16], the mixing parameters [3,17,18], and the baking parameters [9,19,20] on the densification behavior of the carbon paste. However, few articles have been published on the mechanical behavior of the carbon paste under cyclic compaction loads. Thibodeau et al. performed quasi-static cyclic tests on the green anode paste at a high temperature and on the dry calcined coke of the size −8+14 US mesh [21]. This study showed that the densification of the green anode paste occurs within three phases: (1) the distance between the aggregates decreases and they get in contact, (2) the aggregates reorientate themselves (through translation and rotation) when the stress further increases, and (3) the brittle aggregates break when the internal stresses exceed their strength. The aggregates’ breakage during this experiment was captured by an acoustic waves detection system and was highlighted by a softening behavior on the stress–strain curves [21]. A study that investigated the behavior of the carbon paste subjected to cyclic loadings was [22]. Quasi-static cyclic tests at different strain amplitudes were carried out on a room-temperature ramming paste. It was shown that decreasing the strain amplitude applied to the carbon paste would lead to a considerable decrease in the axial stress and to an increase in the number of cycles needed to reach the target density [22]. The authors presented the following hypothesis: lower strain amplitudes during cyclic loadings enhance the aggregates rearrangement and the relaxation of pore pressure during the unloading phases. In [6,22], the time dependent behavior of the carbon paste was investigated. Relaxation tests carried out on a room temperature carbon paste at different levels of imposed strains revealed a highly time-dependent behavior on the part of the carbon paste under relaxation loads and a negligeable viscoelastic strain during the recovery phase in comparison with the instantly reversible and permanent strains [22]. In [6], a modified creep test was performed on the green anode paste at high temperature. A significant increase in deformation during the creep phase was indicative of a time-dependent behavior for the green anode paste. In [22], vibro-compaction tests done with a maximum amplitude of 1 MPa and at frequencies ranging from 0.1 Hz to 7 Hz. indicated that increasing the frequency within the studied range would lead to an increase in the number of cycles and a decrease in the total time needed to reach the target density The frequency, however, would not affect the final rigidity of the vibro-compacted sample.

The potential effects of the vibro-compaction parameters (e.g., frequency, the duration of maximum load) on the final quality of the compacted green anode paste went under investigation in [23,24,25]. It was found that increasing the vibration frequency and the maximum load applied to the green anode paste would result in increasing the apparent density of the carbon block [23,24,25]. However, Increasing the vibro-compaction time beyond an optimal time would result in a decrease in the apparent density and a deterioration in the mechanical properties of the carbon block [24,25].

The findings of the present work aimed to gain a deeper understanding of carbon paste behavior when it is subjected to cyclic loadings. The study focused on aspects not attended to by similar studies; namely, (1) the effect of the strain rate during cyclic tests on the permanent strain evolution, (2) the effect of the stress amplitude during cyclic tests on the permanent strain evolution, (3) the effect of the strain rate on the shape of the hysteresis generated during the cyclic loading, (4) the effect of the stress amplitude on the hysteresis shape, and (5) the effect of the unloading level at each cycle during cyclic loading on the densification behavior. To this end, an experimental campaign was carried out, consisting of (1) cyclic tests at different strain rates and different maximum stress amplitudes, and (2) cyclic tests at different unloading levels.

Moreover, the test results can be used in future studies to develop an appropriate constitutive law for the carbon paste subjected to cyclic loading. The investigation of the strain rate effect will be useful to determine whether the effect of time should be taken into account in the intended behavior law. In addition, the dependence of the mechanical properties of the carbon paste on the density, the cycle number, and the maximum stress amplitude will be determined. The latter result can be used to suggest empirical formulas for the mechanical properties of the carbon paste.

## 2. Material and Set-Up

### 2.1. Material

The carbon paste tested in this study was a room-temperature ramming paste provided by the industrial partner Alcoa in a ready-to-use formula. According to the provider’s instructions, it is preserved in hermetic containers to prevent the loss of volatiles. The carbon paste is made of calcined anthracite, coal tar pitch, and a softener. The softener decreases the viscosity of the coal tar pitch allowing the formation of the paste at room temperature. For confidentiality reasons, the detailed recipe and the commercial trade name of the carbon paste cannot be disclosed. 

### 2.2. Experimental Set-Up

A Dartec compression apparatus is used to perform the experimental tests (Figure S1), and the axial stress is applied by a computer-controlled servo-hydraulic machine (DARTEC LTD, Stourbridge, UK). The press was modified in the past, the original controller was replaced by a MTS FlexTest 40 (MTS Headquarters, Eden Prairie, MN, USA), and the original load cell by a 250 kN MTS (model 661.22H-01, (MTS Headquarters, Eden Prairie, MN, USA)). The carbon paste is placed in a thin-walled stainless steel mold which has a height of 140 mm, a diameter of 253 mm, and a thickness of 0.3 mm. The Young modulus and the Poisson ratio of the mold were determined by a simple traction test, and they are equal respectively to E = 175 GPa and υ = 0.265. The Mold is equipped with 4 axial and 4 radial strain gauges. They are placed in pairs at a height of 40 mm from the base of the mold and are equally spaced on the mold’s circumference. A calibrated LVDT (range: +40 mm, resolution: 0.001 mm) is placed vertically between the base of the mold and the top of the loading plate to continuously measure the height of the tested samples. Each test (T1 to T8) was repeated twice. The results of the repetitions were similar. Therefore, for each test, only the results of one trial were reported.

## 3. Methodology

To prepare a sample, 6 kg of the carbon paste was placed in a loose state in the mold. The initial height of the samples was approximately 135 mm. Two series of experiments were carried out to investigate the effects of the strain rate, the stress amplitude, and the unloading level on the mechanical behavior of the carbon paste subjected to cyclic loads. What follows is a detailed discussion of these experiments and the obtained results. 

### 3.1. Series I: The Effects of Strain Rate and Stress Amplitude 

**Figure 1:**
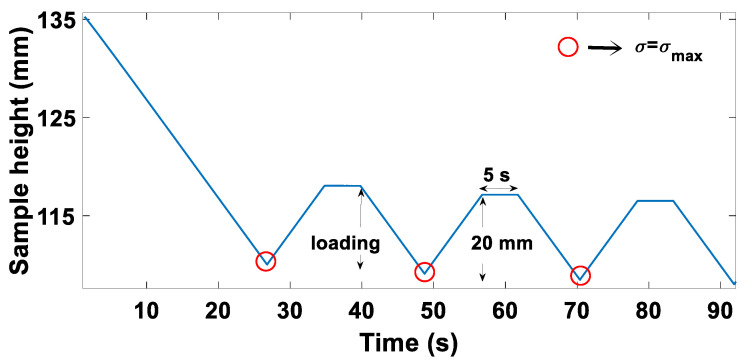
The load path of series I of tests.

This first series of tests consisted of 8 cyclic tests carried out at different strain rates and at different maximum stress amplitudes. The objective was to investigate the effects of the strain rate and those of the maximum stress amplitude on (1) the permanent deformation evolution as a function of the cycle number and (2) the shape of the hysteresis curve generated by the cyclic loading. The load path of these tests is shown in Figure 1. Each cycle consists of three steps. The sample was first compacted at a strain rate $\dot{\epsilon }$ until the vertical stress reaches the maximum stress amplitude specified for the test ($\sigma_{max}$). Then, the sample was unloaded by moving the loading plate upward at the same strain rate of loading for a total relative displacement of 20 mm to ensure a complete unloading of the carbon paste. As the last step, the loading plate was kept fixed for 5 s. The cycling was repeated until the sample would reach a density of approximately 1.6 g/cm^3^. The tests were performed for several values of the strain rate $\dot{\epsilon }$ and the maximum stress amplitude $\sigma_{max}$, which are shown in Table 1. Three values of strain rates were selected at the outset: $\dot{\epsilon }=\;$0.7 × 10^−2^ s^−1^, $\dot{\epsilon }=$ 3.7 × 10^−2^ s^−1^, and $\dot{\epsilon }=$ 7.4 × 10^−2^ s^−1^. The maximum value of $\dot{\epsilon }$ was 7.4 × 10^−2^ s^−1^, which corresponds to a displacement velocity of 10 mm/s. Tests with higher strain rates that approach the values used in the industry could not be carried out because of the hydraulic press capacity limits. It was planned to carry out further tests with additional values of the strain rate within the hydraulic press capacity (<$\dot{\epsilon }=$ 7.4 × 10^−2^ s^−1^). However, it will be shown in the results section that the strain rate in the studied range had no effect on the carbon paste behavior. Therefore, the study of the strain rate effect was limited to the values shown in Table 1. The values of $\sigma_{max}$ were chosen to meet the estimated load level applied to the carbon paste in the industry during cyclic compaction.

### 3.2. Series II: Effects of Unloading Level during Cyclic Tests 

**Figure 2:**
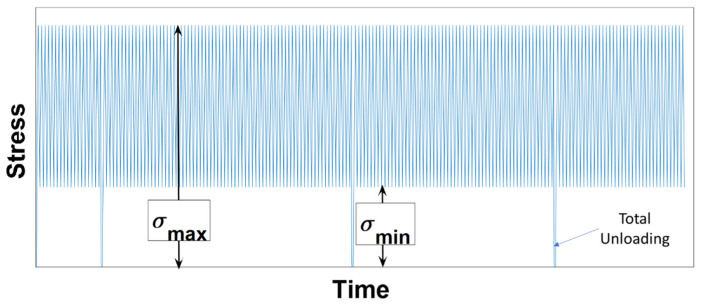
The load path of the second series of tests.

The second series of tests consisted of 5 cyclic compaction tests where the stress amplitude varied between a maximal and a minimal value denoted, respectively, as $\sigma_{max}$ and $\sigma_{min}$ (Figure 2). They were conducted at a constant strain rate: $\dot{\epsilon }=\;$0.7 × 10^−2^ s^−1^. These tests were carried out to investigate the effect(s) of the unloading level on the cyclic behavior of the carbon paste, specifically regarding the permanent deformation evolution in function of the cycle number. The tests were carried out for several values of $\sigma_{max}$ and $\sigma_{min}$, which are shown in Table 2. During tests T-III and T-V, the samples were completely unloaded 3 times to investigate the effect of these complete unloadings on carbon paste densification (Figure 2).

### 3.3. Measurements and Investigated Properties

The axial stress, the sample height and the mold’s local axial and circumferential strains at a height of 40 mm from the mold base were monitored and recorded during all the tests. The measurements were recorded at a time interval depending on the applied strain rate during the test (recalling that each test had a constant strain rate) and having a minimal value of 0.01 s for tests with strain rate $\dot{\epsilon }=0.007\;$s^−1^ and a maximum value of 0.05 s for tests with strain rate $\dot{\epsilon }=0.07\;$s^−1^. The total axial strain of the sample was calculated using the following equation [6]:

(1) $$\epsilon =\frac{h_{0}-h}{h_{0}},$$

where ‘h’ is the sample height and $h_{0}$ is the sample initial height. The Permanent strain at the beginning of cycle ‘*j*’, represented by $\epsilon^{p}\left(j\right)$ is assumed to be equal to the total strain ϵ at this point when the stress is equal to zero. The sample density at the beginning of cycle ‘*j*’ is denoted by ρ(j) and is calculated according to the following equation [6]:

(2) $$\rho \left(j\right)=\frac{m}{\pi r_{0}^{2}h_{0}\left(1-\epsilon^{p}\left(j\right)\right)},$$

where m and $r_{0}$ represent the sample mass and the mold’s initial radius, respectively. The variation of the sample radius during the test is negligeable, thus the initial radius $r_{0}$ is used in the calculation of the density (see Appendix A). The shape of the hysteresis is an important aspect to watch for since its area indicates the energy dissipation during the loading-unloading cycles and its inclination correlates with the sample’s rigidity. For tests T1–T8, the air of the hysteresis w(j) as a function of the cycle number was calculated using the Trapezoidal rule. The focus of this study was to highlight the behavior of the carbon paste during cyclic compaction from the height of 82 mm (ϵ= 0.4, ρ(j)= 1.45 g/cm^3^) to that of 74.5 mm (ϵ= 0.45, ρ(j)= 1.6 g/cm^3^). As a result, all the cycles that resulted in a sample height of more than 82 mm (ρ(j)< 1.45 g/cm^3^) were not considered. When the carbon paste’s density $\rho >1.45\;g/{\mathrm{cm}}^{3}$, the solid skeleton of the carbon paste is already consolidated [21]. The density of the samples before this threshold is so low ($\rho \leq 1.45\;g/{\mathrm{cm}}^{3}$) and the permanent deformation increase during a given cycle is important with respect to the maximum deformation of the cycle. Thus, the reversible and the irreversible deformations could not be differentiated.

## 4. Results and Discussion

### 4.1. Series I

#### 4.1.1. Stress–Strain Behavior of the Carbon Paste Subjected to Cyclic Loading

**Figure 3:**
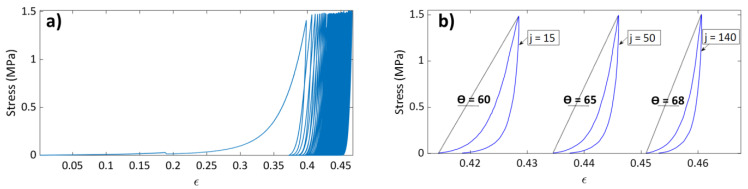
(**a**) Stress–strain curve for test T6, and (**b**) stress–strain curve for cycles 15, 50, and 140 of tests T6.

The stress–strain curve of test T6 (Figure 3a) displays the general behavior of the carbon paste subjected to cyclic loading. The test was carried out under a maximum stress amplitude of $\sigma_{max}=$ 1.5 MPa and with a strain rate of $\dot{\epsilon }=$ 0.007 s^−1^. During the first cycle, the total strain of ϵ= 0.375 (ρ(2)= 1.41 g/cm^3^) (Figure 3a) accounts for 84.84% of the total strain accumulated at the end of the test (ϵ= 0.451). This is due to the fact that the carbon paste is placed in a loose state in the mold, with an initial density as low as $\rho_{0}\;$= 0.88 g/cm^3^. During the subsequent cycles (i.e., cycles 2-145), the strain was increased from ϵ= 0.375 to ϵ= 0.451. Figure 3b shows the hysteresis loop for cycles 15, 50, and 140 of test T6. It is noticeable that the hysteresis area, and therefore the total energy dissipation, decreases as the cycle number increases. The angle between the line that joins the cycle’s start and peak points as well as the strain axis increase when the cycle number increase, which means that the difference between the maximum and the start strains of the cycle declines. These observations suggest that the sample becomes stiffer as the number of cycles increases.

#### 4.1.2. Strain Rate and Stress Amplitude Effect on the Permanent Strain Evolution

**Figure 4:**
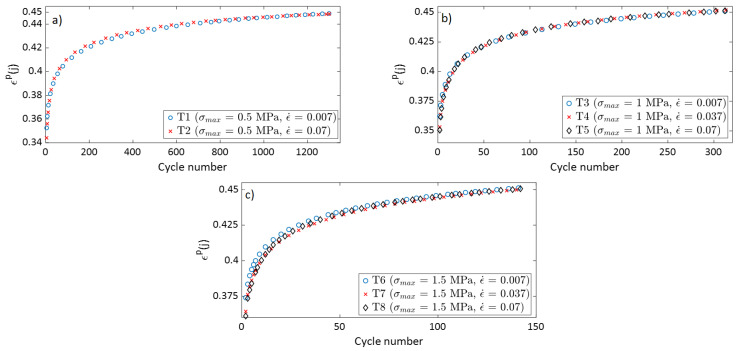
The variation of $\epsilon^{p}$(*j*) in function of the cycle number for: (**a**) T1 and T2; (**b**) T3, T4, and T5; and (**c**) T6, T7, and T8.

To compare the densification behavior of the carbon paste subjected to cyclic loads from tests with different strain rates but subjected to the same maximum stress amplitude, the permanent strain at the beginning of each cycle $\epsilon^{p}\left(j\right)$ was calculated and plotted as a function of the cycle number. The permanent strain can be determined only at the beginning of each cycle, since during loading recoverable and permanent strains cannot be differentiated. Figure 4 shows the variation of $\epsilon^{p}\left(j\right)$ as a function of the cycle number for tests T1 to T8. For all the tests, the permanent strain increased rapidly at the beginning of the test, when the density of the sample was the lowest. As the number of cycles increases, the permanent strain rate decreased. The samples became denser, with additional permanent strains demand more cycles. 

Figure 4 show that tests carried out under the same maximum stress amplitude ($\sigma_{max}$) but with different strain rates had very similar curves of $\epsilon^{p}\left(j\right)$ as a function of the cycle number. Therefore, the strain rate in the studied range had no effect on the permanent strain evolution as a function of the cycle number. The permanent strain is not a time-dependent variable, but rather depends on the cycle number and the maximum stress amplitude applied to the carbon paste.

**Figure 5:**
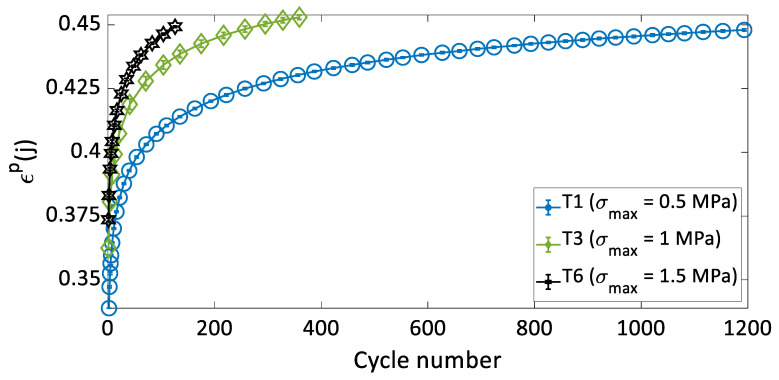
The variation of $\epsilon^{p}$(*j*) in function of the cycle number for tests: T1, T3, and T6 (the error bars in the graph represents the standard deviation of the three performed trials of each test).

To illustrate the effects of the maximum stress amplitude on the densification behavior of the carbon paste, the evolutions of $\epsilon^{p}\left(j\right)$ as a function of the cycle number for tests T1 ($\sigma_{max}=$ 0.5 MPa), T3 ($\sigma_{max}=$ 1 MPa), and T6 ($\sigma_{max}=$ 1.5 MPa) are presented in Figure 5. These tests were done at the same strain rate of $\dot{\epsilon }=$ 0.7 × 10^−2^ s^−1^. The increase in $\sigma_{max}$ led to a decrease the total number of cycles needed to reach the target density of ρ(j)≈ 1.6 g/cm^3^, which corresponds to a permanent strain: namely, $\epsilon^{p}\left(j\right)\approx 0.45$. It is noticeable that there is a substantial difference between the curve representing T1 and the other curves representing T3 and T6. It seems that the stress amplitude of T1 ($\sigma_{max}=$ 0.5 MPa) is below the threshold of stress needed to induce permanent deformation efficiently. The carbon paste is a mixture of a solid skeleton (aggregates) and a binding matrix (liquified coal and fine aggregates). The permanent deformation of this material during the loading-unloading process is controlled by complex mechanisms including aggregate’s rearrangement and the degradation of coarse aggregates into finer particles [21]. Samples compacted at higher stress amplitudes are expected to experience a higher rate of aggregates degradation. Similarly, higher stress amplitudes lead to an increase in the internal stresses during the loading. This internal stress relaxes during the unloading and causes a better densification during next loading. However, the experiments conducted in this study would not allow the quantification of degradation or rearrangement of aggregates. Nor could they determine the cycles during which these mechanisms occur. 

#### 4.1.3. Strain Rate Effect on the Hysteresis Shape

**Figure 6:**
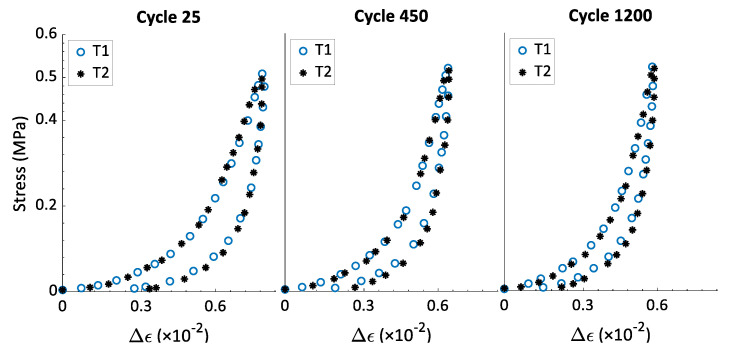
Stress–relative strain curves of cycles 25 ($\epsilon^{p}\left(25\right)=0.39$), 450 ($\epsilon^{p}\left(450\right)=0.43$), and 1200 ($\epsilon^{p}\left(1200\right)=0.45$), from tests T1 and T2 ($\sigma_{max}=$ 0.5 MPa).

**Figure 7:**
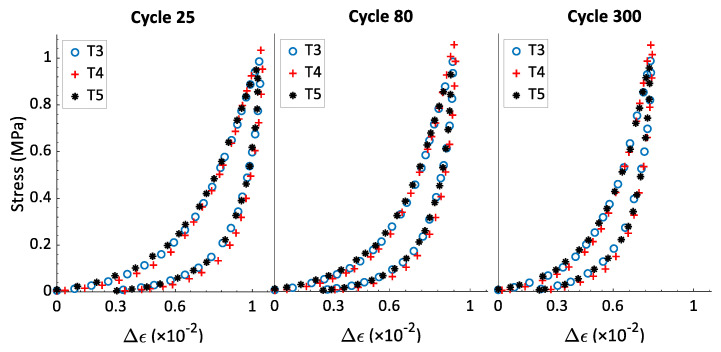
Stress–relative strain of cycles 25 ($\epsilon^{p}\left(25\right)=0.41$), 80 ($\epsilon^{p}\left(80\right)=0.43$), and 300 ($\epsilon^{p}\left(300\right)=0.45$), from tests T3, T4, and T5 ($\sigma_{max}=$ 1 MPa).

**Figure 8:**
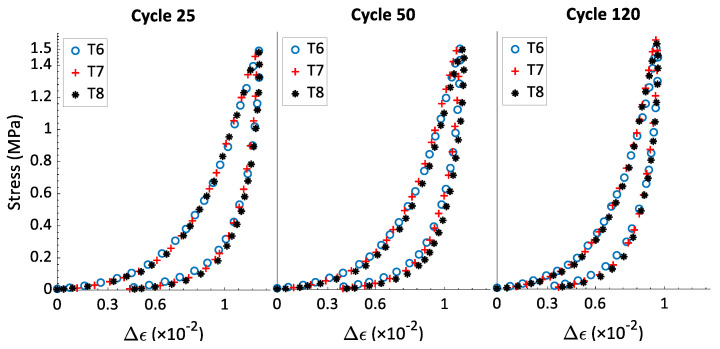
Stress–relative strain curves for cycles 25 ($\epsilon^{p}\left(25\right)=0.42$), 50 ($\epsilon^{p}\left(50\right)=0.43$), and 120 ($\epsilon^{p}\left(120\right)=0.45$), from tests T6, T7, and T8 ($\sigma_{max}=$ 1.5 MPa).

The hysteresis shape reveals information about some mechanical properties of the material. Its area represents the dissipated energy during the loading-unloading caused by the material internal friction or the material viscosity. Likewise, its inclination is related to the sample stiffness. As the material becomes stiffer, its inclination with respect to the strain axis will become less significant. To compare the effects of the strain rate on the hysteresis shape, the relative strain ∆ϵ has been calculated. For any cycle ‘*j*’, the relative strain is defined as $∆\epsilon =\epsilon -\epsilon^{p}$(*j*), which represents the increment of strain during the cycle ‘*j*’ with a strain origin being set to zero at the beginning of each cycle. The aim of this presentation is comparing the hysteresis slope and width of cycles across different tests with different values for $\epsilon^{p}$(*j*). Figure 6 shows the stress–relative strain curves for cycles 25, 450, and 1200 of tests T1 and T2, both with $\sigma_{max}=$ 0.5 MPa. One can notice that the strain rate value does not affect the hysteresis shape. The same observation can be made in Figure 7 and Figure 8 for tests T3–T8.

**Figure 9:**
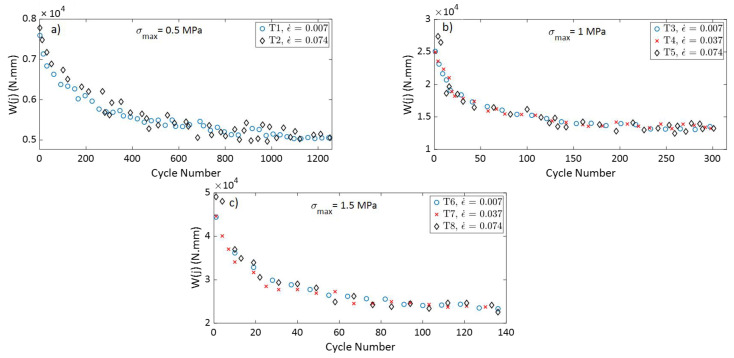
The variation of *w*(*j*) in function of the cycle number for: (**a**) T1 and T2; (**b**) T3, T4, and T5; and (**c**) T6, T7, and T8.

Figure 9 presents the variation of the hysteresis area w(j) as a function of all cycles of the first series of tests (i.e., T1–T8). This information supports the results provided in Figure 6, Figure 7 and Figure 8 and represents the data on the hysteresis shape for all cycles of the first series of tests. For all the tests, the value of w(j) decreases when the number of cycle increases; the rate of change is highest at the beginning of the test but decreases to a minimal value at the end of the test. The tests that have the same value of $\sigma_{max}$ and different values of the strain rate $\dot{\epsilon }$ have similar curves of w(j) vs. cycle number. In this way, the strain rate does not affect the hysteresis shape, which means it has no effect on the energy dissipation and the evolution of the rigidity during cyclic tests with a constant maximum amplitude. In [22], rigidity tests were carried out on compacted carbon paste samples. The samples were vibro-compacted under an axial stress amplitude of 1 MPa, and with frequencies of 0.1 Hz, 2 Hz, and 4Hz to reach a final density of 1.6 g/cm^3^. The results of the rigidity tests showed that all the sample had approximately the same rigidity, with the frequency in the studied range having no effect on the final rigidity of the compacted carbon paste. The observations from Figure 6 and Figure 9 thus corroborate the results presented in [22] regarding the effect of the strain rate (or of the frequency) on the rigidity of a sample compacted under cyclic loading with a constant maximum stress. 

#### 4.1.4. Effect of the Stress Amplitude on the Hysteresis Shape

**Figure 10:**
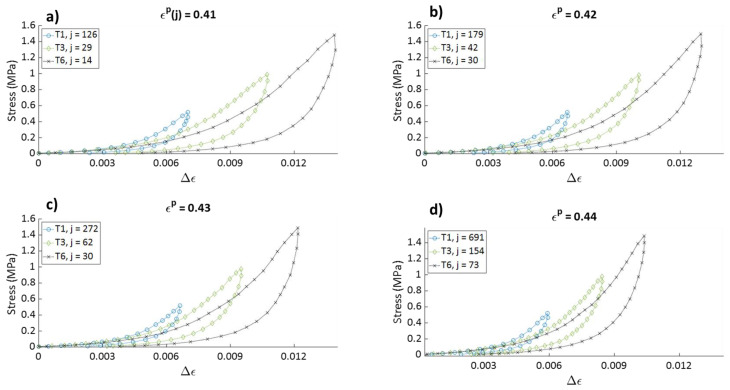
Stress–relative strain curves of cycles: (**a**) 126 of T1, 23 of T3 and 14 of T6; ((**b**) 179 of T1, 42 of T3, and 30 of T6; (**c**) 272 of T1, 62 of T3, and 30 of T6; and (**d**) 691 of T1, 154 of T3, and 73 of T6.

To assess the effect of the stress amplitude on the mechanical behavior of the carbon paste, the stress–relative strain curves of cycles from tests T1 ($\sigma_{max}=$ 0.5 MPa), T3 ($\sigma_{max}=$ 1 MPa), and T6 ($\sigma_{max}=$ 1.5 MPa), all having approximately the same values of $\epsilon^{p}\left(j\right)$ are plotted in Figure 10. For the same value of $\epsilon^{p}\left(j\right),$ the stress–relative strain curve has a steeper slope during loading when the cycle belongs to a test with a lower maximum stress amplitude $\sigma_{max}$. This means that the sample compacted to reach a target density will be stiffer when the maximum stress amplitude is lower. This observation could be related to the phenomenon of aggregates crushing. It could be suggested that two samples that have the same densification level, but different stress–relative strain relations have different aggregates crushing levels caused by the application of different stress levels. It is likely that higher stress levels cause a higher crushing of the coarse aggregates, which can lower the sample stiffness.

#### 4.1.5. Effect of the Cycle Number on the Hysteresis Shape

**Figure 11:**
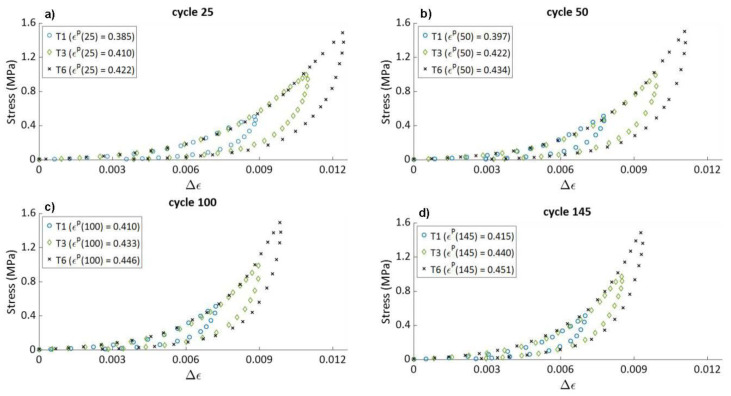
Stress–relative strain curves of (**a**) cycle 25, (**b**) cycle 50, (**c**) cycle 100, and (**d**) cycle 145 of tests T1, T3, and T8.

Figure 11 shows the stress–relative strain curves for cycles 25, 50, 100, and 145 for tests T1 ($\sigma_{max}=$0.5 MPa), T3 ($\sigma_{max}=$1 MPa), and T6 ($\sigma_{max}=$ 1.5 MPa). For the same cycle number of tests T1, T3, and T6 the stress–relative strain curves during loading are superimposed. As Figure 5 shows, for any cycle number ‘*j*’, the value of $\epsilon^{p}\left(j\right)$ increases with a higher $\sigma_{max}$. Therefore, a sample that was compacted at a low stress amplitude and which had a lower compaction degree (lower $\epsilon^{p}\left(j\right)$) at a definite cycle number ‘*j*’ had the same stiffness during loading as a sample compacted with a higher stress amplitude and having a higher compaction degree (higher $\epsilon^{p}\left(j\right)$) at the same cycle ‘*j*’. It can be concluded, therefore, that the elastic properties of the material during the loading phase depend on the cycle number and not on the sample’s apparent density. 

### 4.2. Series II

#### Effect of the Unloading Level on the Permanent Deformation 

**Figure 12:**
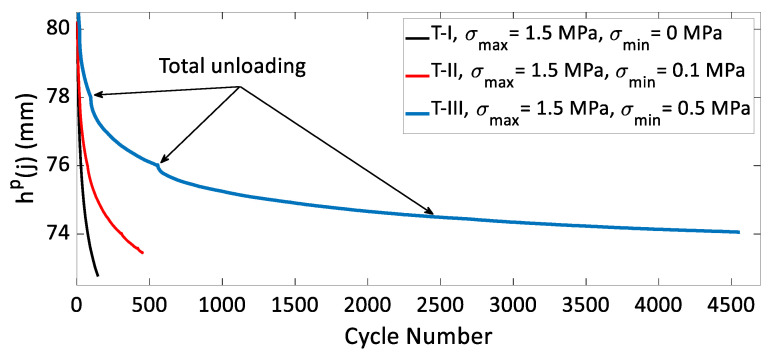
The variation of $h^{p}\left(j\right)$ in function of the cycle number for T-I, T-II, and T-III.

**Figure 13:**
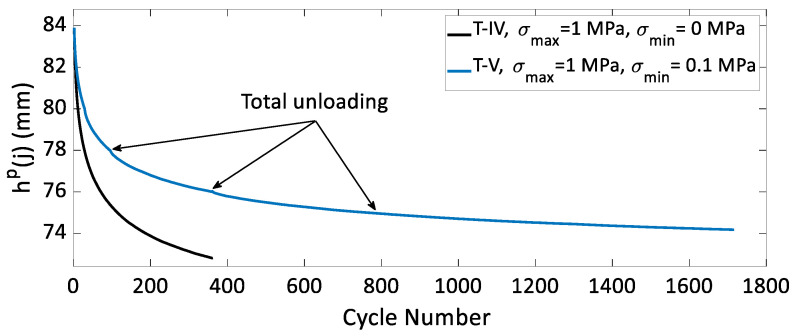
The variation of $h^{p}\left(j\right)$ in function of the cycle number for T-IV and T-V.

To investigate the effect(s) of the unloading level during cycling on carbon paste’s behavior, cyclic tests with complete and partial unloadings were conducted according to the load path shown in Figure 2. The values of the maximal and the minimal stress applied in these tests are identified in Table 2. Since in some of these tests the samples were not completely unloaded ($\sigma_{min}\neq 0$), $\epsilon^{p}\left(j\right)$ could not be measured. To compare the densification behavior of the carbon paste across several tests with the same value for $\sigma_{max}$, the value for the sample height at the stress peaks, represented by $h^{p}\left(j\right)$, was plotted as a function of the cycle number in Figure 12 for tests T-I, T-II, and T-III and in Figure 13 for tests T-IV and T-V. At the same cycle number, the value of $h^{p}\left(j\right)$ is lower when the minimal stress amplitude $\sigma_{min}$ is higher. Recall that three complete unloadings were performed during tests T-III and T-V. The purpose of these unloadings was to demonstrate the effect of a complete unloading during cyclic compaction tests on the densification evolution. These two Figures also indicate an enhancement of densification reflected by a slope change in the curve of $h^{p}\left(j\right)$ vs cycle number for tests T-III and T-V when the samples are fully unloaded. It can be inferred from these data that a complete unloading at each cycle leads to a better densification of the carbon paste during the next cycle. Complete unloading at the end of the cycle decreases the contact forces between the aggregates and give them the possibility to rearrange into a more densified configuration over the next loading. These results, corroborate those obtained for asphalt mixtures in [26].

## 5. Conclusions

In this paper, a specific room-temperature carbon paste used in the aluminum industry was experimentally tested to investigate any potential effects of the strain rate, maximum stress magnitude, and unloading level on its behavior during cyclic loading. The results obtained provide a framework for the development of an appropriate numerical model to reproduce the behavior of carbon paste under the cyclic loading. The experimental results and their implications for modelling the compaction process of carbon paste will be delineated/outlined below.

1. The evolution of the permanent deformation as a function of the cycle number during cyclic tests with a constant maximum stress amplitude is nonlinear and is not influenced by the strain rate. Therefore, the law representing the irreversible behavior of carbon paste subjected to cyclic loading must be independent of time. In addition, the law itself or its parameters must consider the non-linearity of the permanent deformation as a function of the number of cycles.
2. During cyclic tests, the stress–relative strain hysteresis curves changed shape as the number of cycles increased. They become narrower and gain a steeper slope. This reflects the evolution of the elastic properties of the paste as the number of cycles increases. On the other hand, the hysteresis shape of the stress–relative strain curves is not influenced by the strain rate. The part of behavior law describing the reversible behavior of the carbon paste subjected to cyclic loading must be independent of time and should include variable elastic parameters that evolve during compaction.
3. Carbon paste samples with the same density that were subjected to different maximum stress amplitudes during compaction had different stiffness values, as highlighted by different slopes of the stress–relative strain hysteresis curves. This result implies that the elastic properties of a compacted carbon paste do not depend exclusively on its density. Therefore, the elastic parameters of any law intended to represent the cyclic behavior of carbon paste should not be exclusively as a function of the density.
4. Carbon paste samples that had been subjected to the same number of cycles but different maximum stress amplitudes in their loading histories had different densities but the same elastic properties. This is reflected by the same stress–relative strain curve during loading (Figure 11). Therefore, the evolution of the elastic properties of carbon paste during cyclic compaction can be defined as a function of the cycle number.
5. Complete unloading at the end of each cycle during cyclic compaction of the carbon paste enhanced the accumulation of the permanent deformation. This behavioral aspect must be taken into consideration in any work aiming at modeling the shaping process of carbon paste.

In a nutshell, when describing elastic processes during cyclic loading, any behavior law proposed for carbon paste subjected to cyclic compaction must include time-independent elastic parameters that evolve as a function of the cycle number. If irreversible processes during cyclic loading are to be described, the formulated behavior law should be nonlinear and time independent. As a final recommendation, it is suggested that cyclic tests be carried out on the carbon paste with strain rates that approach those used in the industry.

## Figures and Tables

**Table 1:** The values of $\sigma_{max}$ and $\dot{\epsilon }$ for series I tests.

Test	T1	T2	T3	T4	T5	T6	T7	T8
$\sigma_{max}$ (MPa)	0.50		1.00			1.50		
$\dot{\epsilon }$ (s^−1^) × 10^−2^	0.7	7.4	0.7	3.7	7.4	0.7	3.7	7.4

**Table 2:** The values of $\sigma_{max}$ and $\sigma_{min}$ of the second series of tests.

Test	T-I	T-II	T-III	T-IV	T-V
$\sigma_{max}$ (MPa)	1.5	1.5	1.5	1	1
$\sigma_{min}$ (MPa)	0	0.1	0.5	0	0.1

**Figure A1:**
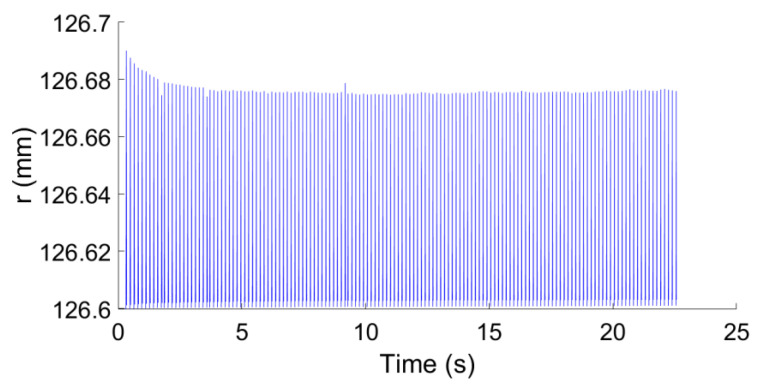
The variation of the sample radius in function of time for test T6.

## Data Availability

There is no data to report.

## Supplementary Materials

The following are available online at https://www.mdpi.com/article/10.3390/ma15031263/s1, Figure S1: Experimental set-up.

## Appendix A

The variation in the sample radius as a function of time while doing test T6 is represented in Figure A1. The variation in the sample’s radius is equal to that in the mold’s radius since the sample and the mold are in constant contact. Test T6 is the one with the highest value of $\sigma_{max}=$ 1.5 MPa, and thus has the highest value of radial deformation. The radius of the sample at the end of each cycle returns to its initial value $r_{0}=$ 126.6 mm. Thus, to calculate the sample density at the beginning of the cycle, the initial radius can be used. The maximum variation of the radius during T6 is ∆r= 0.09 mm, which is negligeable with respect to the axial strain while running the test ($\epsilon^{p}\left(1\right)\approx$ 0.375).
