# Supplementary material for: Strain Rate and Stress Amplitude Effects on the Mechanical Behavior of Carbon Paste Used in the Hall–Héroult Process and Subjected to Cyclic Loadings
Source: Materials (Basel). 2022 Feb 8;15(3):1263. doi: 10.3390/ma15031263 (PMC8839192; doi:10.3390/ma15031263)
Supplement: Supplementary file 1 [file materials-15-01263-s001.zip › materials-1454924-supplementary.pdf]

Supplementary Materials

Strain Rate and Stress Amplitude Effects on The Mechanical Behavior of Carbon Paste Used in The Hall–Héroult Process and Subjected to Cyclic Loadings

Zahraa Kansoun [1,*], Hicham Chaouki [2], Donald Picard [3], Julien Lauzon-Gauthier [4], Houshang Alamdari [5] and Mario Fafard [1]

1 Department of Civil and Water Engineering, NSERC/Alcoa Industrial Research Chair MACE3, Aluminium Research Centre—REGAL, Université Laval, Quebec, QC G1V 0A6, Canada; mario.fafard.2@ulaval.ca
2 SAFI Quality Software Inc., Quebec, QC G1X 1S7, Canada; hicham.chaouki.1@ulaval.ca
3 Eddify Technologies Company, Quebec, QC G1P 0B3, Canada; dpicard@eddyfi.com
4 Continuous Improvement Smelting Technology, Alcoa, Deschambault-Grondines, QC G0A 1S0, Canada; julien.lauzon-gauthier@alcoa.com
5 Department of Mining, Metallurgical and Materials Engineering, NSERC/Alcoa Industrial Research Chair MACE3, Aluminium Research Centre—REGAL, Université Laval, Quebec, QC G1V 0A6, Canada; houshang.Alamdari@gmn.ulaval.ca
* Correspondence: zahraa.kansoun.1@ulaval.ca; Tel.: +15816812007

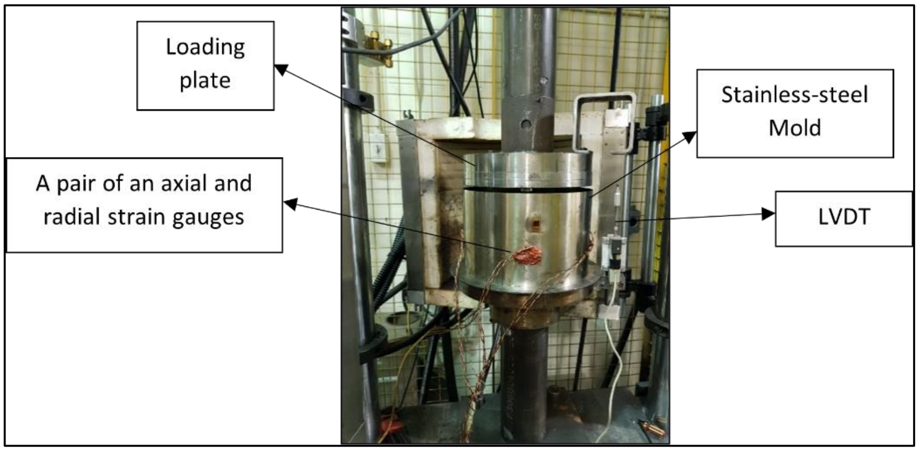

Figure S1. Experimental set-up.
